# Predictors of survival of natural killer/T-cell lymphoma, nasal type, in a non-Asian population: a single cancer centre experience

**DOI:** 10.3332/ecancer.2016.688

**Published:** 2016-11-02

**Authors:** Jule Vásquez, Mariana Serrano, Lourdes Lopez, Cristian Pacheco, Shirley Quintana

**Affiliations:** Department of Medical Oncology, Instituto Nacional de Enfermedades Neoplásicas, de Enfermedades Neoplásicas, Lima 34, Perú

**Keywords:** survival, predictors, natural killer/T-cell lymphoma, chemotherapy

## Abstract

**Background:**

Natural killer/T-cell lymphoma (NKTCL), part of T-cell and NK-cell neoplasms in the World Health Organisation (WHO) classification, is an aggressive lymphoma with poor prognosis more predominantly seen in Asian and South American countries. This study evaluates the factors associated with survival among patients with newly diagnosed NKTCL in Peru.

**Methods:**

Information was abstracted from medical records (MR) for all NKTCL patients >13 years of age at the Instituto Nacional de Enfermedades Neoplasicas (INEN) between 2002 and 2011. The estimate of the survival curves was performed by the Kaplan-Meier method, and the difference was computed by the log-rank test.

**Results:**

Around 226 MR were reviewed, 153 met the selection criteria, the median age was 40 years (14–84). The median progression-free survival (PFS) was 20 months, five year PFS was 42.6%, univariable analysis (UA) showed statistical significance (p < 0.05) for male sex, non-nasal primary site, advanced clinical stages, B symptoms, poor performance status, regional nodal involvement (RNI). In the multivariate analysis the only poor prognostic factors was primary non-nasal (Hazard ratio (HR) = 2.40, 95% confidence interval (CI) = 1.43– 4.02, P = 0.01). The median overall survival (OS) was 49 months, five year OS was 48.9%, UA showed statistical significance for non-nasal primary site, advanced clinical stages, B symptoms, lactate dehydrogenase (LDH) > normal, RNI and local tumour invasion. In the multivariate analysis, primary non-nasal was the only poor prognostic factor with HR = 2.57, 95% CI = 1.37–4.83, P = 0.03.

**Conclusions:**

In Peru, OS of NKTCL is similar to other countries. This result suggests that non-nasal NKTCL is the only poor prognostic factor of OS and PFS.

## Introduction

Natural killer/T-cell lymphoma (NKTCL) is a rare haematological malignancy which is typically extranodal, and it has two main subtypes: nasal and nasal-type. It is characterised by prominent necrosis and cytotoxic phenotype associated with the Epstein-Barr virus [1]. NKTCL is more prevalent in Asia, Central, and South America [2–9]. The upper aerodigestive tract (nasal cavity, nasopharynx, paranasal sinuses, palate) is commonly involved, with the nasal cavity as the prototype. Extranodal sites of involvement include the skin, soft tissue, and testicles [3, 10–14]. Some cases may also be accompanied by secondary nodal involvement [15].

The survival of NKTCL is poor [13]. Adverse prognostic factors associated with worse survival have been described, such as non-nasal primary, clinical staging, nodal involvement, Ki-67 expression, the International Prognostic Index (IPI), the Korean Prognostic Index (KPI), large cells, local tumour invasiness, and circulating EBV-DNA levels among others [16–25]. However, in our country (Peru), there are no studies assessing prognosis factors in these patients.

The aim of this retrospective study is to describe predictors of OS and PFS as well as clinical and pathological features of patients with NKTCL treated at our centre.

## Methods

### Patients

The study population included all newly diagnosed patients >13 years of age with a pathological confirmation of NKTCL seen at the National Institute of Neoplastic Diseases (INEN) in Lima,Peru between January 2002 and December 2011. Patients with other previous cancers, positive serology for HIV, and diagnosis of aggressive NK cell leukaemia, or incomplete MRs were excluded. The diagnosis of NKTCL was based on the WHO 2008 classification of haematopoietic and lymphoid tissues [1]. All cases were reviewed by an expert panel in lymphomas to confirm diagnosis.

### Laboratory findings and staging

Haematological tests, including complete blood count, liver, and renal function tests, and LDH were performed. Local tumour invasion was defined differently according to the two subtypes. The nasal NKTL was defined in accordance with 7th ed., 2010 TNM classification of the American Joint Committee of Cancer. Any nasal NKTCL with T3 or greater were considered as local invasive in the analysis. For non-nasal NKTCL, the definition of local invasiveness differed according to primary sites. For gastrointestinal NKTCL, local invasiveness referred to T4 lesion in TNM system. In NKTCL primarily involving soft tissue such as muscle or skin, invasion of neurovascular structure or bone invasion was considered as local invasion. Regional nodal involvement was defined as the invasion of lymph nodes corresponding to N1, N2, or N3 of the primary lesion based upon TNM staging system. Accordingly, M1 nodes at TNM system were not categorised as regional lymph nodes in the analysis [16].

The staging was based on modified System Cotswolds Ann Arbor [26]. Performance status was evaluated according to the Eastern Cooperative Oncology Group (ECOG) scale [27]. The response was evaluated based on the revised response criteria for lymphomas [28].

### Statistical analysis

A descriptive analysis of the information through frequencies, percentages, and measures of central tendency were performed. OS was defined as the time from the date of diagnosis to date of last visit or death from any cause, and PFS from the initiation of treatment to disease relapse/progression, last follow-up or death from any cause, whichever occurred first. The overall and PFS were estimated with the Kaplan-Meier method and differences were tested using the log-rank test. The Cox proportional hazard models were used to identify predictors of survival of NK/T-cell lymphomas. A level of p <0.05 was considered for a statistical significance. The multivariate analysis was performed with all factors with statistical significance in the univariate analysis. SPSS version 22.0 was used for statistical analysis.

## Results

### Patient characteristics

A total of 226 patients were seen at our centre during the study period, according to the database of the Department of Epidemiology, 212 records were retrieved, 37 did not receive chemotherapy, 15 had previous treatment, 5 were younger than 13 years, and 2 had metacronic neoplasms. Finally 153 cases were reviewed for analysis. This study was approved by the Institutional Review Board at our institution. The clinical characteristics of the 153 patients are outlined in Table 1. The majority of the patients were primary nasal (126, 82.4%). The non-nasal primary (27, 17.4%) included primary lesions at the following sites: Waldeyer’s ring (n = 5), skin (n = 5), oropharynx (n = 3), hard palate (n = 3), soft tissue (n = 3), splenic (n = 1), tongue (n = 1), gastrointestinal tract (n = 1), alveolar ridge (n = 1), cervical lymph node (n = 1), inguinal lymph node (n = 1), larynx (n = 1), and hypopharynx (1).

### Treatment

Radiotherapy only was the treatment used in 79 patients (51.6%), chemotherapy only in 25 (16.3%), chemotherapy followed by radiotherapy in 33 (21.6%), radiotherapy followed by chemotherapy in 14 (9.2%), and concurrent chemoradiotherapy in 2 (1.3%) (Table 1). The early stages were treated with radiotherapy only in 77 cases (57.5%) (nasal = 73), chemotherapy only in 11 (8.2%) (nasal = 9), chemotherapy followed by radiotherapy in 32 cases (23.9%) (nasal = 22), radiotherapy followed by chemotherapy in 12 (8.9%) (nasal = 11), concurrent chemoradiotherapy in 2 (1.5%) (nasal = 2). None of these patients underwent haematopoietic stem cell transplantation. The advanced stages were treated with radiotherapy only in two cases (10.5%) (nasal = 1), chemotherapy only in 14 (73.7%) (nasal = 6), and chemotherapy followed by radiotherapy in one case (5.3%) (non-nasal = 1), radiotherapy followed by chemotherapy in two cases (10.5%) (nasal = 2).

Chemotherapy regimen used was cyclophosphamide, doxorubicin, vincristine, and prednisone (CHOP) in seven patients (early stages n = 2 , advanced stages n = 5) and CHOEP-14 (CHOP + etoposide) in 18 (early stages n = 9) patients. No one patient received SMILE regimen (dexamethasone, methotrexate, ifosfamide, l-asparaginase, and etoposide) as it was included as a salvage treatment just at the end of 2013. The chemotherapy regimens used either as monotherapy, prior, concurrent, or followed by radiotherapy were CHOP in 33 (44.5%), CHOEP-14 in 40 (54.1%), and other regimen in one case (1.4%), totalling 74 patients with this type of treatment.

Twenty-five patients received chemotherapy only (early stages n = 11), ranging the number of cycles of chemotherapy between 1 and 8 with a median of 2.5 cycles. Among the patients in early stages receiving chemotherapy followed by radiotherapy with number of cycles ranging between 1 and 8, we find 69.6% (23 out of 33) received six cycles. It was also noted that only one patient with advanced disease received this modality of treatment.

### Treatment response

The early stages had response rates as follows: CR n = 92 (68.7% ) (stage I = 68, stage II = 24) PR n = 16 (11.9%) (stage I = 10, Stage II = 6), SD n = 3 (2.2%) (stage I = 2, stage II = 1), PD n = 23 (17.2%) (stage I = 11, stage II = 12). The advanced diseases had response rates as follows: CR n = 7 (36.8%) (stageIII = 1, stage IV = 6), PR n = 5 (26.4%) (stage IV = 5), SD n = 0, PD n = 7 (36.8%) (stage III = 1, stage IV = 6).

### Progression-free survival

**Figure 1.**
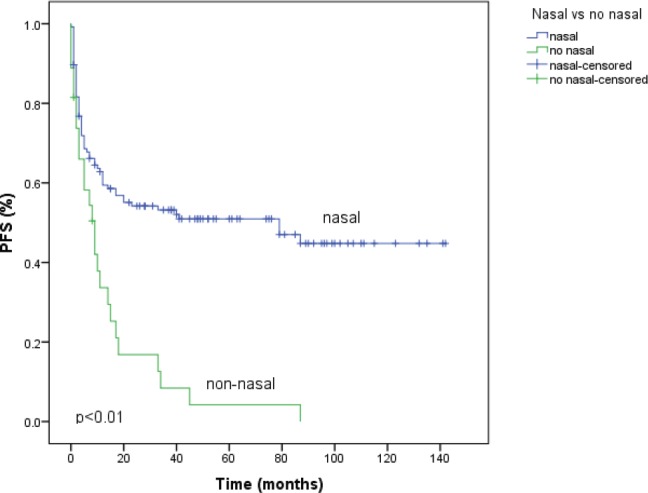
Progression-free survival (PFS) according to primary site of natural killer/T-cell lymphoma. Primary nasal (n = 126) and non-nasal (n = 27).

With a median follow-up time of 18 months, the median PFS time was 20 months (95% CI 0–41), and the five year PFS rate was 42.6%. The variables that showed statistical significance towards a worse outcome in the univariate analysis were male sex, non-nasal primary site, advanced clinical stages, presence of B symptoms, poor performance status (PS), and nodal involvement. The median PFS for the nasal primary was 79 months (95% CI 14–143) and the five year PFS was 50.9% as shown in Figure 1 and Table 2. The six variables with statistical significance were studied by multivariate analysis, showing that the only independent poor prognostic factor was primary non-nasal site (HR = 2.40, 95% CI = 1.43–4.02, P = 0.01) (Table 2).

### Overall survival

**Figure 2.**
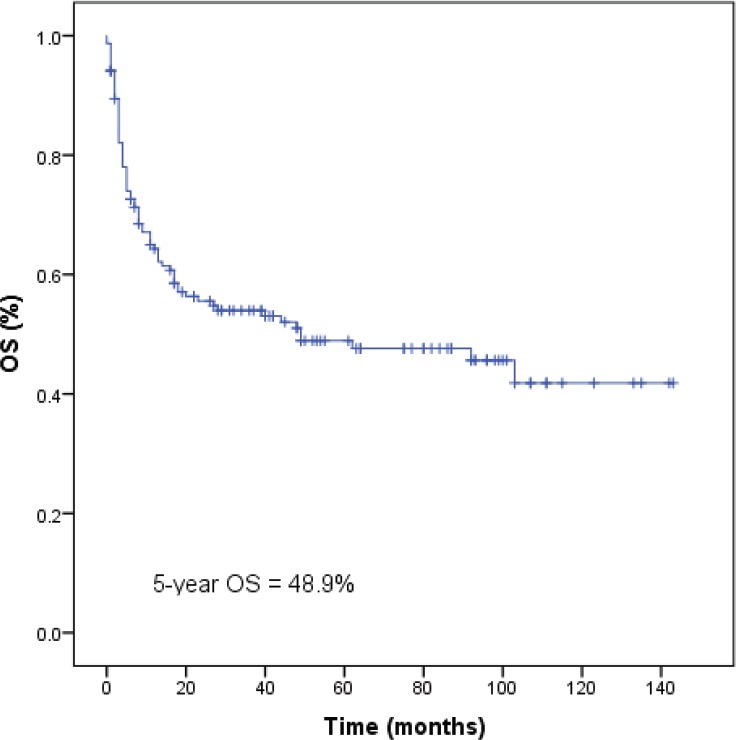
Survival of 153 natural killer/T-cell lymphoma patients. OS, overall survival.

**Figure 3.**
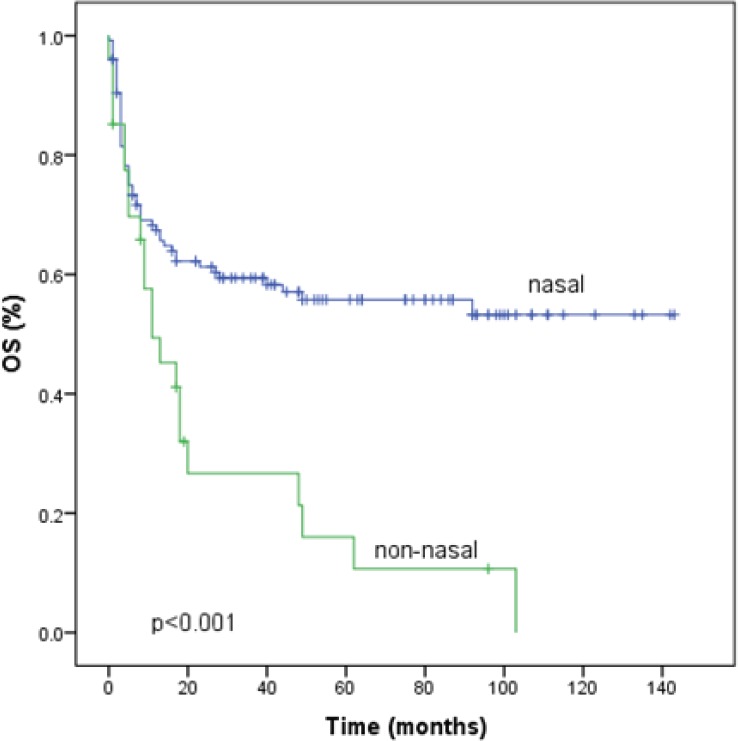
Survival according to primary site of natural killer/T-cell lymphoma. OS, overall survival.

Seventy-five patients (49%) had died by the time of the analysis. With a median follow-up of 48 months (1–143 months). The median OS time was 49 months (95% CI 0–98) and the five year OS rate was 48.9% (Figure 2). The factors that showed statistical significance towards a worse outcome in the univariate analysis were non-nasal primary site, advanced clinical stage, presence of B symptoms, increased LDH, nodal involvement and local tumour invasion. For nasal primary the median OS time was not reached and the five year OS rate was 55.8% as shown in Table 3 and Figure 3. The multivariate analysis showed that the only poor prognostic factor was primary non-nasal site (HR = 2.57, 95% CI = 1.37–4.83, P = 0.03) (Table 3).

We performed a sub-analysis for early disease and we found the median OS for nasal primary (117, 87.3%) was not reached and that for non-nasal primary (17, 12.6%) was 17 months, p = 0.003. In the multivariate analysis we identified three factors for poor prognosis, non-nasal site (HR = 3.1, 95% CI 1.50–6.37 p = 0.002), presence of B symptoms (HR = 1.86, 95% CI 1.037–3.34, p 0.037), and DHL > normal (HR = 1.84, 95% CI 1.04–3.27, p = 0.035).

## Discussion

We present here 153 NKTCL patients who fulfilled our selection criteria diagnosed over a period of ten years. To the best of our knowledge it is the largest Latin American study in NKTCL patients [4–8] and the second largest in the whole American continent [9–12]. The number of patients were similar to some seen in multicentre studies [2, 13, 15, 20–22, 32–36]. The present study shows that the clinical characteristics of nasal NKTCL in Peru are similar to those described in other Latin American as well as Asian and Western countries [2, 3, 6, 8, 9, 29, 30]. The treatment used in our population was according to the clinical staging, with radiotherapy only or concurrent with chemotherapy for early stages, and chemotherapy only for advanced disease, as is described in the international literature [31]. L-asparaginase-based regimen (SMILE treatment) was not part of the treatment, as this was incorporated in our institution just in 2013 [37].

In this series, the PFS was 20 months which is similar to reported by Huang *et al* at 18 months [21]. However, the five year PFS was 29.8% which is lower than our study where the five year PFS was 42.6%. This discrepancy might be because of a lower proportion of patients with presence of B symptoms in our study (36% versus 54%, respectively) and also could have influenced the higher percentage of patients with early stages in our study (83% versus 79.5%, respectively) . In contrast, Lee *et al* [16] showed a five year relapse-free survival (RFS) of 60%. This result is different because this was calculated on patients who had achieved complete remission.

In our study, we show that patients with NKTCL have poor survival, with a median OS of 49 months. In other studies, the median OS has ranged between 30 and 50 months [2, 16, 21, 34]. However, when we consider the survival for non-nasal NKTCL this was nine months, different from data reported by Au *et al* [3] where extranasal cases had a median OS of four months. This was probably because 19% of those patients did not receive treatment because of advanced disease unlike our study where all patients received treatment.

Regarding the cumulative probability of survival at five years, our study shows a 49% OS rate, whereas in other studies it ranged between 39% and 50%. [13, 16, 21, 34, 37, 38]. When evaluating the non-nasal primary this was 16% which is comparable to the study of Au *et al* [3]. Only a non-nasal primary site was an independent adverse predictor in the multivariate analysis which we find is also the same variable identified in other studies [3, 12, 16, 32]. The primary site of this type of lymphoma had been evaluated previously but with the term upper aerodigestive tract NKTCL. This included lymphomas confined to nasal cavity, nasopharynx, larynx, pharynx, and oral cavity [16]. In our study nasal type refers only to nasal cavity.

In our study, we found that the primary site is more important than clinical stage as independent prognostic risk factor, and this finding was reported by Au *et al*’s study. Even non-nasal primary with apparently localised disease had poor prognosis [3]. The biological distinction between these two subgroups remains unknown, hence necessitating future studies with genetic and epigenetic profiling.

Other prognostic factors have been evaluated in NKTCL patients such as Ki-67 expression, EBV viral load, lymphocyte and monocyte counts [19, 21, 25, 33]. In the last years c-Myc expression, beta-2 microglobulin, CD30-CD38 expression, the albumin to globulin ratio [38–44], CD56 expression, higher levels of HLA-DR negative, CD33, CD11b myeloid-derived suppressor cells (MDSCs), CD14 monocytic MDSCs, independent adverse prognostic scores have also been used for evaluation [45]. However, in our study we could not evaluate these factors either because of incomplete baseline data or because of lack of tests for these factors in our centre.

## Conclusion

In conclusion, in Peru the OS for NK/T-cell lymphoma is similar to other Latin American as well as Asian countries. The results suggest that non-nasal NKTCL is the only poor prognostic factor of OS and PFS which we find is even more important than the clinical staging itself. This poor prognostic factor is seen in early stages as well. It is important to conduct multicentre prospective studies including the most important clinical, laboratorial, pathological, and viral prognostic factors in order to make an accurate prognostic index.

## Conflict of interest

The authors declare no competing financial interests.

## Figures and Tables

**Table 1.** Clinical and pathological characteristics.

Characteristics	N	%
Age, years, Median/range	40/[14–84]	
Age intervals ≤60 years old/>60 years old	131/22	85.6/14.4
Sex (male/female)	98/55	64.1/35.9
Primary site Nasal/Non-nasal	126/27	82.4/17.4
Stage Early (I, II) Advanced (III, IV)	134 (91/43) 19 (2/17)	87.6 (59.5/28.1) 12.4 (1.3/11.1)
B Symptoms Yes/No	55/98	35.9/64.1
LDH >Normal/<Normal/No available	58/83/12	37.9/54.2/7.8
Performance Status (ECOG) (1,2,3,4)	138/15/0/0	90.2/9.8/0/0
Regional nodal involvement Yes/No	60/93	39.2/60.8
Local tumour invasion Yes/no	44/109	28.8/71.2
Type of treatment		
Chemotherapy only	25	16.3
Radiotherapy only	79	51.6
Chemo-radiotherapy	49	32.1

**Table 2.** PFS according to patients features and univariate/multivariate analysis of prognostic factors.

Characteristics	N	Median, months	Five year survival rate, %	Univariate analysis	Multivariate analysis	
				p	HR (95% CI)	p
Age, years						
≤60	131	17	41.9			
>60	22	40	45.5	0.376		
Sex (male/Female)	98/55	12/79	37.5/51.3	0.033		
Primary site						
Nasal	126	79	50.9		2.40 (1.43–4.02)	0.01
Non-nasal	27	9	4.2	<0.001		
Stage						
Early (I–II)	134	33	46.7			
Advanced (III–IV)	19	7	14	0.002		
B symptoms						
Yes	55	7	33.2			
No	98	40	50.6	0.016		
PS						
1	138	33	44.5			
2	15	4	26.7	0.028		
LDH						
>normal	58	11	38.1			
≤normal	83	45	50.0	0.060		
Regional nodal involvement						
Yes	60	7	27.9			
No	93	79	51.9	0.005		
Local tumour invasion Yes/No	44/109	12/40	31.7/46.9	0.083		

NR = Not reached, PFS = progression-free survival

**Table 3.** Overall survival, according to patient features and univariate/multivariate analysis of prognostic factors.

Characteristics	N	Median, months	Five year survival rate, %	Univariate analysis	Multivariate analysis	
				p	HR (95% CI)	p
≤60	131	49	49			
>60	22	49	48.3	0.572		
Sex (male/female)	98/55	40/NR^(*)^	45.8/54.1	0.106		
Primary site						
Nasal	126	NR^(*)^	55.8			
Non-nasal	27	11	16.0	<0.001	2.57 (1.37–4.83)	0.03
Stage						
Early (I–II)	134	92	52.8			
Advance (III–IV)	19	8	21.1	0.005		
B Symptoms (yes/no)	55/98	13/103	37.6/55.2	0.014		
PS						
1	138	62	50.1			
2	15	8	40.0	0.160		
LDH						
>normal	58	17	43.8			
≤normal	83	NR^(*)^	57.9	0.018		
Regional nodal involvement						
Yes	60	11	35.4			
No	93	NR^(*)^	57.5	0.002		
Local tumour invasion						
Yes	44	13	39.3			
No	109	103	52.5	0.040		

(*) Not reached

## References

[1] Swerdlow SH (2008). WHO Classification of tumours of haematopoietic and lymphoid tissues. International Agency for Research on Cancer (IARC).

[2] Xu PP (2012). Prognostic factors of Chinese patients with T/NK-cell lymphoma: a single institution study of 170 patients. Med Oncol.

[3] Au WY (2009). Clinical differences between nasal and extranasal natural killer/T-cell lymphoma: a study of 136 cases from the International Peripheral T-Cell Lymphoma Project. Blood.

[4] Arber DA (1993). Nasal lymphomas in Peru: High incidence of T-cell immunophenotype and Epstein-Barr virus infection. Am J Surg Pathol.

[5] Quintanilla-Martinez L (1999). Histological and immunophenotypic profile of nasal NK/T cell lymphomas from Peru: high prevalence of p53 overexpression. Hum Pathol.

[6] Cabrera ME (2007). Nasal natural killer/T-cell lymphoma and its association with type ‘‘i’’/XhoI loss strain Epstein–Barr virus in Chile. J Clin Pathol.

[7] Barrionuevo C (2007). Extranodal NK/T-cell lymphoma, nasal type: study of clinicopathologic and prognosis factors in a series of 78 cases from Peru. Appl Immunohistochem Mol Morphol.

[8] Gualco G (2011). Clinicopathologic and molecular features of 122 Brazilian cases of nodal and extranodal NK/T-cell lymphoma, nasal type, with EBV subtyping analysis. Am J Surg Pathol.

[9] Van de Rijn M (1997). Extranodal head and neck lymphomas in Guatemala: high frequency of Epstein-Barr Virus-associated sinonasal lymphomas. Hum Pathol.

[10] Khan L (2015). A single institution experience of extranodal natural killer/T cell lymphoma of nasal type. Leuk Lymphoma.

[11] Avilés A (2015). Nasal NK/T-cell lymphoma. A comparative analysis of a Mexican population with the other populations of Latin-America. Mediterr J Hematol Infect Dis.

[12] Li S (2013). Extranodal NK/T-cell lymphoma, nasal type: a report of 73 Cases at MD Anderson Cancer Center. Am J Surg Pathol.

[13] Vose J, Armitage J, Weisenburger D (2008). International T-Cell Lymphoma Project. International peripheral T-cell and natural killer/T-cell lymphoma study: pathology findings and clinical outcomes. J Clin Oncol.

[14] Ahn HK (2012). Extranodal natural killer/T-cell lymphoma from skin or soft tissue: suggestion of treatment from multinational retrospective analysis. Ann Oncol.

[15] Li YX (2008). Clinical features and treatment outcome of nasal-type NK/T-cell lymphoma of Waldeyer ring. Blood.

[16] Lee J (2006). Extranodal natural killer T-cell lymphoma, nasal-type: a prognostic model from a retrospective multicenter study. J Clin Oncol.

[17] Kohrt H, Lee M, Advani R (2010). Risk stratification in extranodal natural killer/T-cell lymphoma. Expert Rev Anticancer Ther.

[18] Li YJ (2013). The Glasgow Prognostic Score (GPS) as a novel and significant predictor of extranodal natural killer/T-cell lymphoma, nasal type. Am. J Hematol.

[19] Jiang L1 (2014). Prognostic significance of Ki-67 antigen expression in extranodal natural killer/T-cell lymphoma, nasal type. Med Oncol.

[20] Huang JJ (2012). A novel prognostic model for extranodal natural killer/T-cell lymphoma. Med Oncol.

[21] Huang JJ (2011). Absolute lymphocyte count is a novel prognostic indicator in extranodal natural killer/T-cell lymphoma, nasal type. Ann Oncol.

[22] Chen KL (2015). The prognostic nutritional index predicts survival for patients with extranodal natural killer/T cell lymphoma, nasal type.

[23] Kim TM (2005). Local tumor invasiveness is more predictive of survival than International Prognostic Index in stage IE/IIE extranodal NK/T-cell lymphoma, nasal type. Blood.

[24] Chim CS (2004). Primary nasal natural killer cell lymphoma: long-term treatment outcome and relationship with the International Prognostic Index. Blood.

[25] Hsieh PP (2007). EBV Viral Load in tumor tissue is an important prognostic indicator for nasal NK/T-cell lymphoma. Am J Clin Pathol.

[26] Lister TA (1989). Report of a committee convened to discuss the evaluation and staging of patients with Hodgkin’s disease: Costwolds meeting. J Clin Oncol.

[27] Oken MM (1982). Toxicity and response criteria of the Eastern Cooperative Oncology Group. Am J Clin Oncol.

[28] Cheson BD (1999). Report of an international workshop to standardize response criteria for non-Hodgkin’s lymphomas. NCI Sponsored International Working Group. J Clin Oncol.

[29] Pagano L (2006). NK/T-cell lymphomas ‘nasal type’: an Italian multicentric retrospective survey. Ann Oncol.

[30] McKelvie PA, Thompson PA, Tam CS (2012). Peripheral T cell and natural killer (NK) T cell lymphomas: a clinicopathological study from a single Australian centre. Histopathology.

[31] Chaudhary Rk, Bhatt VR, Vose JM (2015). Management of extranodal natural killer/T-cell lymphoma, nasal type. Clin Lymphoma, Myeloma Leuk.

[32] Suzuki R (2010). Prognostic factors for mature natural killer (NK) cell neoplasms: aggressive NK cell leukemia and extranodal NK cell lymphoma, nasal type. Ann Oncol.

[33] Huang JJ (2013). Prognostic significance of peripheral monocyte count in patients with extranodal natural killer/T-cell lymphoma. BMC Cancer.

[34] Cai Q (2013). Fasting blood glucose is a novel prognostic indicator for extranodal natural killer/T-cell lymphoma, nasal type. Br J Cancer.

[35] Cai Q (2014). New prognostic model for extranodal natural killer/T cell lymphoma, nasal type. Ann Hematol.

[36] Watanabe T (2010). Pretreatment total serum protein is a significant prognostic factor for the outcome of patients with peripheral T/natural killer-cell lymphomas. Leuk Lymphoma.

[37] Kwong YL (2012). SMILE for natural killer/T-cell lymphoma: analysis of safety and efficacy from the Asia Lymphoma Study Group. Blood.

[38] Huang X (2014). Both c-Myc and Ki-67 expression are predictive markers in patients with Extranodal NK/T-cell lymphoma, nasal type: a retrospective study in China. Pathol Res Pract.

[39] Yoo C (2014). Prognostic impact of beta-2 microglobulin in patients with extranodal natural killer/T cell lymphoma. Ann Hematol.

[40] Kim WY (2015). Prognostic implications of CD30 expression in extranodal natural killer/T-cell lymphoma according to treatment modalities. Leuk Lymphoma.

[41] Wang L (2015). CD38 expression predicts poor prognosis and might be a potential therapy target in extranodal NK/T cell lymphoma, nasal type. Ann Hematol.

[42] Bi XW (2016). The pretreatment albumin to globulin ratio predicts survival in patients with natural killer/T-cell lymphoma. PeerJ.

[43] Kim SJ (2016). A prognostic index for natural killer cell lymphoma after non-anthracycline-based treatment: a multicentre, retrospective analysis. Lancet Oncol.

[44] Liang R (2016). Natural killer/T cell lymphoma, nasal type: a retrospective clinical analysis in North-Western China. Oncol Res Treat.

[45] Zhang H (2015). Myeloid-derived suppressor cells inhibit T cell proliferation in human extranodal NK/T cell lymphoma: a novel prognostic indicator. Cancer Immunol Immunother.

